# Review of Bionanocomposite Coating Films and Their Applications

**DOI:** 10.3390/polym8070246

**Published:** 2016-06-29

**Authors:** Mhd Abd Cader Mhd Haniffa, Yern Chee Ching, Luqman Chuah Abdullah, Sin Chew Poh, Cheng Hock Chuah

**Affiliations:** 1 Department of Mechanical Engineering, Faculty of Engineering, University of Malaya, Kuala Lumpur 50603, Malaysia; mmmhaniff@yahoo.com.sg (M.A.C.M.H.); pohsc@um.edu.my (S.C.P.); 2 Department of Chemistry, Faculty of Science, University of Malaya, Kuala Lumpur 50603, Malaysia; chchuah@um.edu.my; 3 Department of Chemical Engineering, Faculty of Engineering, University Putra Malaysia, Serdang 43400, Malaysia; chuah@upm.edu.my; 4 Institute of Tropical Forestry and Forest Product (INTROP), University Putra Malaysia, Serdang 43400, Malaysia

**Keywords:** bionanocomposite, coating film, crosslink, nanomaterials, biodegradable polymers

## Abstract

The properties of a composite material depend on its constituent materials such as natural biopolymers or synthetic biodegradable polymers and inorganic or organic nanomaterials or nano-scale minerals. The significance of bio-based and synthetic polymers and their drawbacks on coating film application is currently being discussed in research papers and articles. Properties and applications vary for each novel synthetic bio-based material, and a number of such materials have been fabricated in recent years. This review provides an in-depth discussion on the properties and applications of biopolymer-based nanocomposite coating films. Recent works and articles are cited in this paper. These citations are ubiquitous in the development of novel bionanocomposites and their applications.

## 1. Introduction

Bionanocomposite coating films are composite materials that consist of natural or synthetic biodegradable polymers and nano-scale materials. Bionanocomposites are known as a novel class of advanced materials. In these materials, the polymer matrix, which includes natural or synthetic polymers or biomolecules, is considered the biological origin, whereas nano-scale materials are regarded as value-added materials. Composite materials exhibit stronger physical, chemical and mechanical properties than their constituent materials. However, conventional composite materials mechanically differ from nanocomposites because of their exceptionally high surface-to-volume ratio. Nanocomposites are incorporated with a large variety of systems, including organic and/or inorganic materials as the general class and either one of the phases with one, two, or three dimensions less than 100 nm. In recent years, researchers and industries have been moving toward developing bio-based nanocomposites to address environmental issues and to find alternative sources for petroleum-based chemicals. The results of recent studies show that bionanocomposites are excellent green technology materials with good biodegradability, biocompatible properties, and the capability to mimic bio materials. Consequently, bionanocomposites have been ubiquitous in numerous applications, including coating films, which have been realized via novel and conventional research and technologies.

Natural or synthetic biodegradable polymers play a major role in the fabrication of bionanocomposite coating films incorporated with nano-scale materials. The properties and applications of a bionanocomposite coating film depends on the characteristic of the nano-scale materials. Several natural polymers have been categorized in previous studies, and each of these materials inherently possesses different characteristics, such as molecular arrangement, active functional groups, bonding nature, thermal behavior, and solubility. Apart from inorganic or synthetic organic compounds, some minerals and clay minerals are also used as nano-scale materials to fabricate bionanocomposite coating films. These materials are utilized in various interdisciplinary fields such as bio-inspired materials, bio-mineralization processes, and biomimetic systems [1]. Furthermore, the properties of a bionanocomposite coating films depend on the characteristics of the biopolymers, the stoichiometric ratio of the constituent materials, and the cross-linking among the constituent materials as well as on the biopolymer macromolecular matrix. The applications of bionanocomposite coating films have been ubiquitous in different industries, such as aerospace, food, biomedical, tissue engineering, paint, packaging, and glass coating.

This paper aims to review studies on polymer-based nanocomposite coating films and their applications. Furthermore, the UV-blocking properties of biopolymers and recently fabricated bionanocomposite coating films are comprehensively discussed. Film characterizations are presented as well.

## 2. Constituent Materials of Bionanocomposite Coating Films

Natural polymers are found in renewable resources such as cellulose, wood fiber, starch, chitosan, pullulan, alginate, protein, shellac, lignin, and polyhydroxyalkanotes. They are used in many industrial and biomedical applications such as in preparing implant devices, biomimetic materials, films, coating materials [2], insulation materials, paints, paint inks, biodegradable packing materials, adhesives, footwear components, synthetic leathers, lubricants, biodiesel, and plasticizers [3]. In addition, main class synthetic biodegradable polymers, including polyglycolide, polylactic acid, polybutylene succinate and poly(vinyl alcohol) (PVOH) and other biodegradable polyurethanes, are reviewed in this paper. Types of nanoscale materials, their significance and loading level into a polymer matrix as well as an effect of nanoscale materials on coating application are discussed. Moreover, a range of applications and properties of film-forming materials are investigated in this review.

### 2.1. Natural Polymers

Six natural polymers, such as plant cellulose, lignin, chitosan, pullulan, polyhydroxyalkanoate, and protein are discussed in this review based on their source of origin, chemical structure, functional group, potential surface modifications, and their applications.

#### 2.1.1. Cellulose

**Figure 1:**
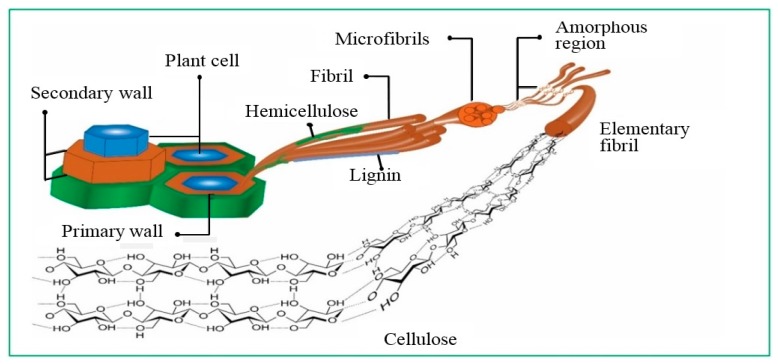
Graphical illustration of the hierarchical structure of cellulose extracted from plants.

Cellulose is one of the major natural and renewable biopolymer resources. It is extensively used in fabricating advanced polymer-based nanocomposite materials in the form of cellulose nanocrystals (CNCs), and is adopted in the sustainable production of materials on an industrial scale [4]. Cellulose exists as a cellular hierarchical biocomposite in all wood and plant materials incorporated in other materials such as lignin, hemicelluloses, waxes, extracts and trace elements [5]. A graphical illustration of cellulose is provided in Figure 1. The molecular arrangement and bonding nature of cellulose in plant cell wall have been clearly presented by Habibi et al. [6]. CNCs are well-ordered crystalline structures within cellulose fibers which is composed of amorphous and crystalline regions. CNCs are formed by the breakup of microfibrils into shorter crystalline parts. CNCs have been mainly referred to as microcrystals, whiskers, microcrystalline, nanofibers, or nanofibrils in recent years. Different mechanical and chemical treatments have been used to prepare CNCs. Further individualization of CNCs occur by using cryocrushing, disintegration and defibrillation processes [7].

Considering the hydrogen bond in their crystalline region, CNCs exhibit good strength and insoluble nature in most solvents. The mechanical properties such as the theoretical values of Young’s modulus, tensile strength, and elongation at break modulus of the CNCs of cotton and tunicate were reviewed by Habibi et al. [6]. The mechanical properties of some plant fibers are listed with their values in Table 1.

The cell wall is composed of several complex layered structures with primary and secondary walls. The primary wall has three layers which are covered by the secondary wall. Each layer is composed of microfibrils, and the thick middle layer of the secondary wall determines the mechanical properties of the fibers. The presence of fiber with an optimum quantity determines the mechanical strength of advanced polymer-based nanocomposites. Therefore, understanding the physical, chemical and mechanical properties of natural fiber is a value-added concept to develop improved natural polymer-based nanocomposites. The properties of the natural fibers are not only dependent on weather condition, soil, and climate, but are also affected during the processing of the fiber, such as during retting, scotching, bleaching, and spinning [9].

Cellulose fibers are used as reinforcing materials in several industries because of their high thermal stability, high aspect ratio, relatively high strength, low density, excellent tensile strength, high durability, good mold capability, and high stiffness [10]. Furthermore, these fibers are abundantly available, biodegradable, biocompatible, cheap, renewable, have low abrasive nature, and exhibit good mechanical properties. In addition to these inherent properties, surface modification treatments also enhance the characteristics of CNCs. Such treatments are possible because of the abundance of the hydroxyl group on CNC surface. The results showed that various chemical processes such as silylation [11], esterification, etherification, oxidation, and polymer grafting [6] have successfully functionalized CNCs in recent years.

**Figure 2:**
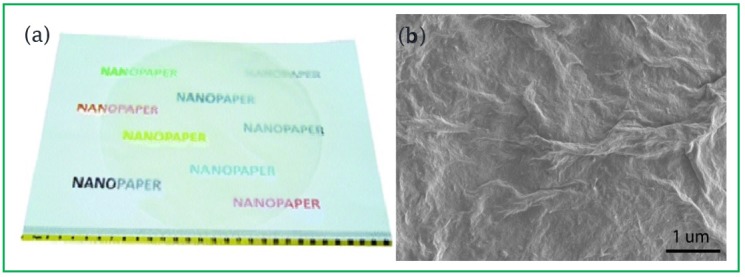
(**a**) Photograph of a 200 mm diameter cellulose nanopaper structure on top of conventional A4 copy paper; (**b**) Scanning electron micrograph of hybrid microfibrillated cellulose nanofibers/montmorillonite nanopaper surface. Reprinted (adapted) with permission from [16], copyright (2016) American Chemical Society.

The archetypal properties and chemical modification possibilities of CNCs have directed the considerable academic and industrial interests toward the potential of these materials in various applications, such as in coating films [12], nanopaper (Figure 2), nanocomposites [13], high-performance materials, biomedicals, catalysts, sensors, electronics, and energy [14,15]. However, CNCs have poor water-vapor barrier capacity because of the huge amount of hydroxyl groups on the nanofibrillated cellulose surface.

#### 2.1.2. Chitosan

Chitosan is an extraordinarily versatile natural polymer. It is known as one of the most promising biopolymers for fabricating advanced materials [17,18]. Next to cellulose, chitosan is the second most abundant polysaccharide found in nature [19,20]. Furthermore, over 100 billion tons of chitosan have been converted annually from living materials through the deacetylation process of chitin. Chitosan is a polyamino-saccharide that consists of active primary amine, primary hydroxyl, and secondary hydroxyl groups on its molecular chain known as β-(1-4)-2-amino-2-deoxy-d-glucose. These unbranched molecular units act as functional monomers, which form the chitosan polymer.

The active primary amine and hydroxyl groups of chitosan allow its structural modification with suitable cross-linking agents. Accordingly, the physical properties of chitosan are enhanced, whereas swelling degree in its aqueous system is reduced [18]. Cross-linking agents for chitosan, such as glyoxal, glutaraldehyde, epichlorohydrin, genipin, ethylene glycol diglycidyl ether, *N,N′*-methylenebisacrylamide, and sulfuric acid, have been intensively reviewed by Xu et al. [18]. They also investigated the cross-linking mechanism, preparation methods, toxicity, hydrophobicity, feasibility, and drawbacks of these agents. Chitosan has been extensively used in several fields, such as in medicine, protein separation and identification, chiral compound separation, and coating field as well as the winemaking industry because of its physical robustness, thermal stability [18], bivalent mineral-chelating capability and antibacterial behavior [21]. However, chitosan has poor mechanical properties, water resistance limit, and gas-barrier capability. Therefore, its application is only possible in the presence of water and humidity [22,23]. Cationic polysaccharide of chitosan are highly sensitive, and thus, heterogeneous materials that exhibit inadequate properties with trace amounts of anionic substances and nanoparticles in the solution are formed [24]. However, countless strategies, such as the addition of plasticizers and salt, the chemical modification of hydroxyl groups, the cross-linking of polysaccharides, the use of suitable solvents, the change of pH, the addition of different polysaccharides, and the blending with other polymers, have been studied to improve the mechanical properties and other properties of chitosan [17,25].

Chitosan is a positively charged polysaccharide used in medical and drug delivery applications [26,27]. Interestingly, a recent novel approach on electrostatic interaction, in which cationic polysaccharides are combined with anionic substances and nanoparticles, has turned the drawback of cationic polysaccharides in an aqueous medium into an advantage to develop bionanocomposites using chitosan as a building block. The results of an ionic system of chitosan can act as a weak polyelectrolyte to generate a charged and non-charged state. The charging of the system can also be manipulated by directly changing the pH value [28].

#### 2.1.3. Lignin

**Figure 3:**
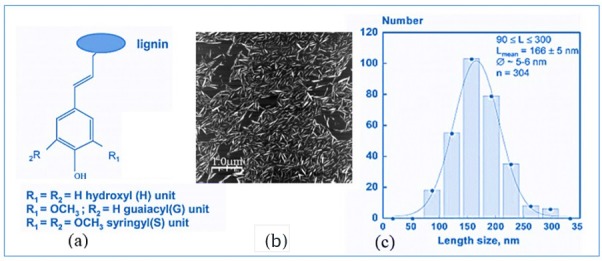
(**a**) The chemical structure of the monomer units found in lignin; (**b**) AFM image of cellulose nanocrystals after acid hydrolysis and (**c**) the average length of the monomer unit found in lignin. Reprinted (adapted) with permission from [31], copyright (2016) American Chemical Society.

Lignin is the second-most abundant natural renewable biopolymer derived from plant materials. In addition, over 70 million tons of lignin is derived annually for different purposes. In 2002, 95% of lignin was used in the form of energy [28] and only 1%–2% was used in industrial products [29]. Lignin exhibits variability in terms of building units and functional groups, including ether and ester linkages, aliphatic and aromatic hydroxyl groups, and methyl groups [30]. Furthermore, aromaticity and bonding nature of the lignin with polysaccharides in the plant cell wall have been discussed by Hambardzumyan et al. [31]. Figure 3 describes the molecular organization, functional groups, and length of a molecular unit.

Lignin is not only enhanced by its aromatic nature and functional groups, but also by its considerable potential adhesive, stabilizing, and suspension-forming properties to present a highly reactive nature. Consequently, lignin is regarded as a good additive for developing biodegradable composite materials and also as a stabilizing agent for ceramics and as an effective alumina suspension for advanced material fabrication. In addition, lignin inherently possesses UV-absorbing capability [32] and demonstrates good mechanical resistance, recalcitrance to biodegradation, and hydrophobic properties.

Meanwhile, recently developed chemically modified lignin, such as lignosulfates, kraft lignin, and acetylated lignin, which contain CNCs or commercial derivatives or nanocellulosic polysaccharides, further improved the hydrophobicity, mechanical resistance, and oxygen barrier properties of the materials. Furthermore, a lignin-based nanocomposite incorporated with CNCs improves the surface water resistance and wettability properties of the materials in the presence of Fenton’s reagent (H_2_O_2_ and FeSO_4_) as an initiator. Graft copolymerization is a simple and attractive approach to change the physical and chemical properties of lignin [32]. Several techniques have been used to modify lignin through the graft copolymerization processes, such as irradiation, chemical, and chemo-enzymatic initiation [33]. The limitation of the biodegradability of lignin copolymer incorporated with vinyl monomers is a drawback in most free radical-based graft copolymerization techniques [34].

**Figure 4:**
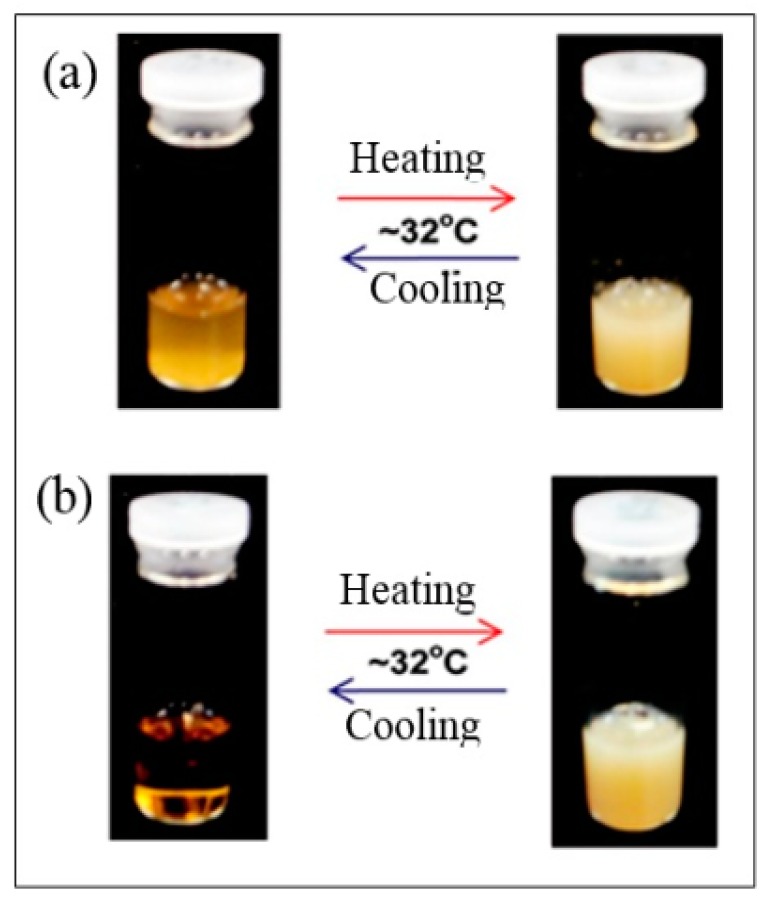
Visual appearance of the phase transition of (**a**) lignin-*g*-poly(*N*-isopropylacrylamide) copolymer and (**b**) lignin-*g*-poly(*N*-isopropylacrylamide) copolymer fully substituted macroinitiator. Reprinted (adapted) with permission from [32], copyright (2016) American Chemical Society.

Consequently, poly-(ε-caprolactone) and l-lactide [35,36] have been invented as alternative monomers to fabricate biodegradable materials from copolymerized lignin. The ring-opening polymerization technique is extensively used to prepare lignin-graft polylactic acid (PLA) and polycaprolactone (PCL) copolymers [37] with a suitable catalyst. The thermal stability and soluble characteristics of lignin and lignin-based copolymers were reported by Kim et al. [32] and illustrated in Figure 4.

#### 2.1.4. Pullulan

**Figure 5:**
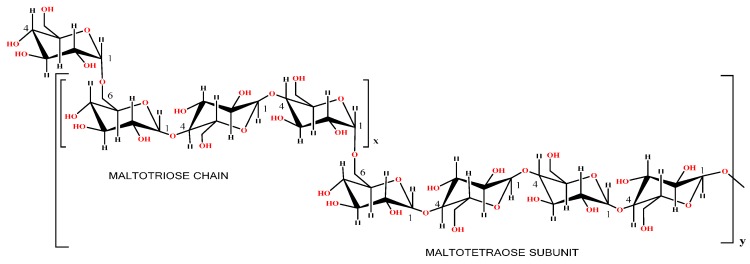
Molecular structure of pullulan with maltotriose and maltotetraose subunit.

Pullulan is another type of natural polymer produced by particular strains of the polymorphic fungus, *Aureobasidium pullulans* as an extracellular, water-soluble polysaccharide. *Aureobasidium pullulans* is a ubiquitous fungus found in environmental samples such as soil and water, particularly as an early-colonizing saprophyte on decaying leaf litter, wood, and many other plant materials, in which they utilize cellobiose but not cellulose [38]. Pullulan is a commercially emerging biopolymer used in diverse industrial applications such as pharmaceutical, chemical, energy production, agriculture, and food industries, among others. It is a linear homopoly-saccharide consisting of regularly repeating maltotriose (or trimer) subunits connected via α-(1→6) glycoside linkage. Maltotriose is a polysaccharide with α-(1→6) linkage with (1→4)-α-d-triglucosides, illustrated in Figure 5. Furthermore, out of the total residue, only 1%–7% of maltotetraose (tetramer) subunits can also be possible in pullulan [39].

The unique pattern of the α-(1→6) linkages among the maltotriose subunits provides distinctive physical properties of pullulan including high water solubility and structural flexibility [39,40]. Consequently, these properties endow the pullulan with physical traits, along with adhesive properties, and enable its capacity for compression, thereby molding strong, oxygen-impermeable films and forming fibers. Leather [41] reported that pullulan fibers and films similar to certain synthetic polymers (plastic derivatives from petroleum) possess oxygen impermeability compared with other polysaccharide films.

Pullulan is highly water soluble, insoluble in organic solvents, non-hydroscopic, and possesses relatively different viscosity than other polysaccharides, i.e., relatively low as compared to most gums, particularly the Arabian gum. Arabian gum has an extremely lower viscosity of ~80–120 cP: with that same concentration, the viscosity of pullulan solution is ~22,000 cP. However, pullulan is significantly less viscous than other gums [42].

Yeast, similar to the form of strain *Aureobasidium pullulans* QM 3090, is the primary producer of pullulan and the production is controlled by the culture medium (pH value) as well as the temperature (optimal temperature ranging from 24 to 32 °C) [43]. Several substrates exist, such as sucrose, glucose, fructose, maltose, starch, or oligosaccharides and others can be used as a substrate for pullulan production. Among them, sucrose is frequently used for this purpose [44].

Several studies have reported on surface modification of pullulan, with the main focus on etherification [45], hydrogenation [46], carboxylation [47], esterification [48], chloroformate activation and succinoylation [49]. Shibata et al. [50] reported a peaceful surface modification of pullulan via phenyl isocyanate (PIC) and hexyl isocyanate (HIC) with clear glass transition temperature (*T*_g_). Hasuda et al. [51] synthesized photo-reactive pullulan using the photo-immobilization technique, and they concluded that photo-reactive pullulan is covalently immobilized on various surfaces; furthermore, pullulan significantly reduces the interaction with protein and cells. Hydrophobically modified pullulan was synthesized by Kuroda et al. [52] using a cholesteryl-bearing pullulan (CHP) and hexadecyl group-bearing pullulan (C16P) via hierarchical self-assembly techniques. Viscosity of the semi-dilute solution (approximately above 2 wt %) of CHP and C16P drastically increased but they formed macroscopic gels at a high concentration [52]. Another study used 3-amminopropyltrimethoxysilane to modify the surface of pullulan via graft-polymerization technology [53].

Pullulan polymer-based nanomaterial and nanocomposite materials have been fabricated in recent years, and they are applied in highly specific fields. In particular, adriamycin-loaded pullulan acetate (PA) and sulfonamide conjugate nanoparticles, as well as PA and oligo-sulfadimethoxine conjugate self-assembled pH-sensitive hydrogel nanoparticles, are used for treating breast tumor cell line (MCF-7), tumor, ischemia and inflammation [54].

Alternatively, pullulan-based bionanocomposite coating materials have been fabricated the first time, incorporated with montmorillonite via the ultrasound-assisted procedure for the exfoliation of inorganic tactoids. Consequently, the coating material exhibits a high oxygen barrier [55].

The pullulan-based sustainable nanocomposite films were characterized by Trovatti et al. [56], with the incorporation of bacterial cellulose. Nonetheless, thermal and mechanical properties of the composite films were enhanced by the nanofibrillated cellulose. Pinto et al. [57] fabricated a transparent nanocomposite thin film based on pullulan polymer incorporated with silver nanoparticles. In addition, pullulan and 6-carboxy pullulan-mediated silver nanoparticles were fabricated by Coseri et al. [58].

#### 2.1.5. Polyhydroxyalkanoate (PHA)

Polyhydroxyalkanoate (PHA) is a linear bio-polymer synthesized by microorganisms including many Gram-positive and Gram-negative bacteria under unbalanced growth conditions for energy storage [59]. Several countries participate in fabricating the PHA worldwide; among them are the USA and China, leading by nearly 50,000 and 10,000 tons per year, respectively. PHA closely resembles the synthetic thermoplastic and most promising biopolymer, possessing complete biodegradability and biocompatibility [60]. Accordingly, PHA is attractive to various applications, such as tissue engineering, packaging, drug delivery, and medical bio-implants.

**Figure 6:**
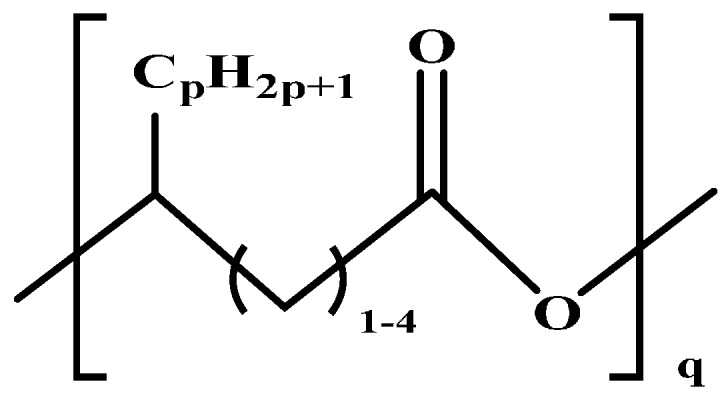
A common chemical structure of polyhydroxyalkanoate monomer, where *p* = 1 to 3; yet *p* = 1 is the most common monomer, 3-hydroxybutyrate; *q* can range from 100 to several thousand [61]. Reprinted (adapted) with permission from [61], copyright (2016) American Chemical Society.

Molecular arrangement of PHA is categorized into two major types of PHA, namely, short-chain length (SCL-PHA) and medium-chain length (MCL-PHA) hydroxyalkonoic acid. These two types are distinguished based on the carbon chain length. Poly (3-hydroxybutyrate) (PHB), poly (3-hydroxyvalerate) (PHV), and their copolymer poly (3-hydroxybutyrate-*C*-hydroxyvalerate) (PHBV) are in the first category, whereas the second category includes the poly (3-hydroxyoctanoate) (PHO) and poly (3-hydroxynonate) (PHN). Furthermore, the MCL-PHA typically contain 3-hydroxyhexanote (HHX), 3-hydroxyheptanoate (HH), and/or 3-hydroxydecanoate (HD) [61,62]. The general formula of PHA monomer is illustrated in Figure 6.

Thermal and mechanical properties of the PHA are commonly expressed in terms of glass to rubber transition (*T*_g_) of the amorphous phase, and this rubbery state of the amorphous phase is generally observed between 19 and 35 °C [63]. However, PHA is a partially crystalline polymer and its melting temperature belongs to the crystalline phase. Moreover, PHB commonly exhibits a degree of crystallinity in the range of 60% to 80%; however, the degree of crystallinity decreases to 30% to 40% as the copolymerization (PHBV) increases (up to 30%). Furthermore, the copolymerization poly(3-hydroxybutyrate-*co-*4-hydroxybutyrate) (PHBHB) exhibits desirable properties in the field of biomedical and agricultural applications [63].

Nonetheless, during the nanocomposite film fabrication, the crystallinity of the PHB was decreased by increasing the amount of nanoparticles. The crystalline size of PHB substantially decreases in PHB-based nanocomposite films compared with that of the neat PHB film incorporated with 1 wt % of the single-wall carbon nanotubes [64].

Some organisms are capable of producing different functionalized PHAs, such as hydroxylated, methylated, brominated, and phenyl derivatives [65]. However, further chemical modification of these functional group-containing polyesters (listed in Table 2) expands the application of PHA in the biomedical field [61]. Furthermore, a step-by-step homogeneity of the chemical reaction allows the tailoring of more useful functional monomers on the PHA surface via graft or block copolymerization technique.

Transesterification is another useful method to modify the PHA surface, in which base or acid catalyst reaction is involved in the modification. Due to the pH sensitivity of the ester group in PHA, the acid-catalyst reaction is more efficient in producing mono-hydroxylated poly(3-hydroxyoctanoate) (PHO) oligomer than the base-catalyst reaction [69]. This finding is explained by the stability of ester bonds of PHO at pH 10 and 12 and the immediate occurrence of hydrolysis when pH was 14. Thus, PHO oligomers form with the unsaturated end group, which may be the result of the *cis*-elimination reaction elucidated with PHB [66].

The chemical modification of PHA via the 3-hydroxybutyrate (3HB) and 4-hydroxybutyrate (4HB) content affords P3/4HB polyester-based polymers toward the high crystallinity from soft elastomers [72]. Conversely, the epoxidation of the unsaturated group in PHA achieves specific physical properties and are highly reactive under mild conditions.

The epoxidized PHA can be used for a cross-linking attachment with bioactive substances as an introduction of the ionizable groups. Chen et al. [73] fabricated poly(3-hydroxybutyrate-*co*-3-hydroxyvalerate) (PHBHV)-based nanocomposite film, incorporated with nano-hydroxyapatite. The hindrance of nanofillers to the mobility of polymer segment results in the film tangent of the loss angle (tanδ). Nonetheless, improved dynamic mechanical properties and activation energy of the films were observed at the maximum load of nano-hydroxyapatite. Furthermore, the obtained nanocomposite films exhibit a slight increment on their glass transition temperature.

#### 2.1.6. Protein

Protein is a natural polymer derived from animals and plants. Collagen, whey protein, casein, egg white, keratin, and fish gelatin are derived from animals, whereas for plant protein, soybean protein, zein (corn protein), and wheat gluten are mainly used in commercial applications [74]. Film-forming ability of protein has been utilized in various industrial applications [75]. Protein films (Table 3) have better oxygen barrier properties and lower water-vapor permeability compared with other nonionic polysaccharide films because of their more polar nature, and more linear (non-ring) structure, and lower free volume [76]. The unique specific interaction capability of these materials with protein DNA, viruses, and other biological structures as well as the accessibility of nanoscale material processing and characterization technique provide a sound method for nanostructured materials in biomedical applications [77].

Surface modification of biomaterials for biomedical applications must satisfy two types of biocompatibility, such that the surface-modified biomaterials elicit the least foreign-body reactions and cell- and tissue-bonding capabilities [78]. Collagen is regarded as one of the most useful biomaterials, a strong candidate for the surface modification of various substrates, exhibiting a number of advantages [79]. The outstanding performance and biomedical application of this protein biomaterial have induced researcher interests toward synthetic fabrication. Jorge et al. [80] reviewed this system in various aspects, such as synthetic routes, characterization, and self-assembly. Surface modification of protein is a promising technique to achieve more suitable derivative for biomedical applications. For example, proteins have been modified with polyethylene glycol (PEG) and monomethoxy polyethylene glycol-based materials, which are biodegradable. Furthermore, the surface-active nucleophilic sites of the protein coupled with activated hydroxyl group of PEG and cyanuric chloride are used to activate the hydroxyl group of PEG. Accordingly, the PEG-based surface modification creates several disadvantages, such as unsuitability for enzyme-containing reactive-SH group on their active site, toxicity of cyanuric acid and its degradation products.

Alternatively, with succinyl succinates of PEG, activated PEG has also been reported as not becoming an inactive-SH group-dependent enzyme. Furthermore, carbonyl dihymidazole active polymer can also bind with lysine amino groups of protein. Nonetheless, to obtain a highly activated polymer is difficult, resulting in low yield of protein binding. Veronese et al. [81] reported an efficient single-step surface modification of protein, via a reaction between monomethoxy polyethylene glycol and 2,4-richloro-phenylchloroformate or *p*-nitrophenylcloroformate.

### 2.2. Synthetic Biodegradable Polymers

Unlike aromatic polymers, aliphatic polyesters are biodegradable and they lack thermal and mechanical properties. Among the biodegradable polymers, aliphatic polyester-based polymeric structure possesses rapid hydrolytic degradation because of the ester functional group in their main chain. Polycondensation and ring-opening polymerization are the major synthetic routes to prepare the biodegradable polyesters. The ring-opening polymerization method is more useful in producing the high molecular weight polymer than the polycondensation of difunctional monomers. However, aliphatic polyesters are nearly the only high molecular weight biodegradable polymers [91]. In biomedical application, poly(glycolic acid), poly(lactic acid) and their copolymers are the mostly used synthetic polymers.

#### 2.2.1. Poly(glycolic acid) (PGA)

Polyglycolide can be obtained via ring-opening polymerization starting with different materials such as cyclic lactone, glycolide (cyclic diester of glycolic acid), and so on. Due to its high degree of crystallization, PGA is hydrophilic and highly degradable but insoluble in most organic solvents except hexafluoroisopropanol [92]. PGA exhibits excellent mechanical properties with high stiffness. Nonetheless, copolymerization of the glycolide with other monomers reduces the stiffness of the resulting fibers [92]. Extrusion, injection, and compression molding as well as the particulate leaching and solvent-casting techniques are used to fabricate PGA-based structures and forms for biomedical application [93].

However, biomedical application of the polyglycolide is limited because of their rapid diacid product form capability. Interestingly, this ability of polyglycolide induced the researchers toward the fabrication of polyglycolide-based surgical sutures.

#### 2.2.2. Polylactide (PLA)

Lactide is a cyclic dimer of lactic acid existing in two stereo isomers signified by dexorotary (D) or levorotary (L). l-Lactide is a natural optical isomer and the dl-lactide is a synthetic blend. Polylactide (PLA) can be elastic or tough, flavor resistant, transparent and can be synthesized via polycondensation, the ring-opening polymerization method or melt-solid polycondensation [94]. During PLA fabrication, d-lactic acid or l-lactic acid can be used as difunctional monomer for polycondensation, whereas d-lactide or l-lactide contribute to produce the PLA via ring-opening polymerization. However, PLA has a higher elongation with lower tensile strength. Furthermore, the significantly high degradation rate of PLA is suitable for drug-delivery systems [92].

The steric-shielding effect of methyl– (–CH_3_) group in PLA enhances its hydrophobic nature than PGA. Depending on the isomer, PLA exhibits different physical properties: l-PLA exhibits a semi-crystalline nature with 37% of crystallinity, whereas dl-PLA is an amorphous polymer. According to the previous studies, degradation depends on the crystalline nature of the polymer materials. Consequently, the degradation order of the PGA and PLA is d,l-PLA < l-PLA < PGA [95]. Consequently, some copolymers of lactide and glycolide have been investigated as bioresorbable implant materials. Moreover, to increase the storage ability of PLA, l-lactide is grafted in the chitosan surface via the ring-opening polymerization technique in the presence of tin catalyst.

#### 2.2.3. Poly(lactide-*co*-glycolide) (PLG)

Glycolide and lactide monomers are used for synthesis of the PLG copolymer, in which monomer ratio and stereo isomers of the lactide are affected on the properties and the applications of the resulting copolymer. The effect includes degradation such that 50 wt % of glycolide and 50 wt % dl-lactide-containing PLG degrade faster than either homopolymer [96]. Furthermore, the range of homopolymer properties that extend the base materials (PGA and PLA) and the binding ability with bioactive ceramics, including bioglass particles and hydroxyapatite, are also attractive for several biomedical applications, including drug delivery, the implant device-making process, and so on [97].

#### 2.2.4. Polybutylene Succinate (PBS)

Polybutylene succinate (PBS) is a member of poly(alkene) dicarboxylate, chemically synthesized via the condensation reaction of succinic acid or adipic acid with ethylene glycol or 1,4-butane diol. PBS exhibits a relatively high melting temperature (*T*_m_ ~90–113 °C) and mechanical properties compared with widely used polyethylene and polypropylene [98]. Furthermore, some other interesting properties were reviewed by Vroman et al. [95]. The electrical properties of the PBS nanocomposite incorporating multi-walled carbon nanotubes (MWNTs) were reported by Lin et al. [99], in which they found the anti-static nature of the nanocomposite material. Viscoelastic behavior and an improved storage modulus of the PBS nanocomposite were further studied, incorporating organically modified montmorillonite. Moreover, PBS exhibits excellent processing ability in the field of textile into melt blown, nonwoven, flat multifilament, monofilament and split yarn fabrics and plastic into injection-molded products. Nonetheless, inadequate gas barrier, melt viscosity, melt strengths and softness characteristics control the PBS for diverse applications.

PBS can further be copolymerized by adipate to increase the rate of biodegradation. Consequently, glycol and aliphatic dicarboxylic acids are used to synthesize the copolymerized poly(butylene succinate-*co*-adipate) (PBSA). PBSA exhibits highly similar properties related to low-density polyethylene and used in various applications, including sheet extrusion, monofilaments, multifilament, laminations, injection-molded cutlery, blow-molded containers, and foam cushion [100].

#### 2.2.5. Poly(vinyl alcohol) (PVOH)

The biodegradation rate of poly(vinyl alcohol) (PVOH) is much higher than the other vinyl polymer, closely related to the poly(enol-ketone). Biodegradation mechanisms of PVOH occur via the oxidation of hydroxyl group followed by hydrolysis and also influenced by the stereo-chemical configuration of the hydroxyl group of PVOH.

PVOH is a partial or complete hydrolysate derivative of the polyvinyl acetate because vinyl alcohol monomer nearly exclusively exists as the tautomeric form acetaldehyde [101]. PVOH is the largest water-soluble polymer synthesized globally because of its higher tensile strength and elongation apart from the oxygen and aroma barrier properties. Furthermore, the water-soluble and reactive characteristics make it a potential material for biomedicine and agriculture as well as for the water-treatment field as flocculants [102] and metal-iron remover [103]. Excellent biodegradable and mechanical properties have made the PVOH an attractive material for biodegradable and disposable plastic substitutes.

#### 2.2.6. Polyurethanes (PUs)

Bio-based polyurethanes (PUs) have received considerable attention in the field of environment-friendly manufacturing processes. The polyol plays a major role in producing urethane linkage in PUs with suitable coupling agents. Interestingly, the use of different polyol results in PUs with different properties through producing polyether- and polyester-based PUs. Polyester-based PUs have been largely applied in coating industries because of their biodegradability, wide range of mechanical strength, low temperature, flexibility, toughness, excellent adhesive property, and chemical and corrosion resistance [104].

Inorganic and organic nanoparticles can be used as coating materials for developing PU-based composites with enhanced properties, and thus, such composites can be applied in coating films [95]. PUs are used in a wide range of industries because of their versatile properties; however, PUs are classified into two major categories, namely, polyether- and polyester-based PUs. PUs can be prepared through either cellular or non-cellular products. Non-cellular PUs may be built from a large number of polyether or polyester polyols by reacting with aliphatic or aromatic diisocyantes. On the one hand, the reaction between ethylene oxide and hydroxide or amine-containing initiators such as sucrose, and glycerol, form polyether-based PU monomers. On the other hand, diacid-containing initiators produce polyester-based PU monomers. The same results can be obtained by substituting monoglyceride derivative from vegetable oil to ethylene oxide [104]. The lack of biodegradable polyether polyol-based PUs has inspired researchers to continue developing bio-based PUs using polyester polyols. Furthermore, polyester polyol has improved hydrolytic stability, low moisture content (less than 0.1%), rapid biodegradable capability, lower acidic number, higher viscosity, higher temperature flexibility, stronger abrasion resistance, and more significant adhesion promotion than polyether polyols, with their enhanced weather capability and strong solvent resistance [105,106]. Alkoxylation and esterification are the most possible chemical routes to synthesize polyols [107]. Nonetheless, polyester polyol has a few drawbacks, such as reduction in the storage stability and hydrolytic resistance, and the water evaporation rate of PU dispersions in UV-curable and adhesive applications.

##### Chemistry of Bio-Based PUs

Polyester polyol is an alcohol with more than two reactive hydroxyl groups per molecule; among their several distinguished properties, biodegradable capability of the polyester-based PUs is important to overcome the environment effect when compared to polyether-based PUs. However, nearly 90% of polyether polyols are used in the production of PUs worldwide in recent years and are derived from petrochemical derivatives such as ethylene and propylene oxides [108].

Consequently, researchers and industries are moving towards producing green technology materials under two aspects: using renewable resources and using biodegradable materials. Numerous studies have reported the increasing urgent demands to replace petrochemical derivatives with renewable resources to overcome global warming and the worsening oil crisis [109]. Vegetable oils are used as renewable resources to synthesize the bio-based polyols through esterification. Epoxidized or derivatives (monoglyceride, diclycerides, etc.) of vegetable oils and diacid initiators are also used to synthesize bio-based polyols. However, several chemical routes have been reported to synthesize polyols from epoxide functional groups using a variety of nucleophiles through the epoxide ring-opening mechanism. Depending on the type of nucleophiles, one or more alcohol-functional derivatives can be added to each aliphatic chain.

The chemical synthetic routes of the epoxide ring-opening have been reported based on the SN1 and SN2 reaction mechanisms. Acid catalyst epoxide ring opening follows the SN1 mechanisms, whereas the reaction between epoxide and strong nucleophiles proceeds through the SN2 mechanisms [107]. Caillol et al. [107] reviewed several synthetic routes for polyol preparation through the epoxide ring opening. Ring-opening routes based on carboxylic acids, such as acetic acid, acrylic acid, fatty acid, hexanoic acid, and octanoic acid, produce polyester polyols with interesting properties, particularly biodegradability and as anti-wear lubricants [110,111].

Polyol-making initiators contribute to producing PUs with different characteristics; these initiators are neither aromatic nor aliphatic diacids. Aromatic diacids, such as isophthalic acid (IPA), phthalic acid, and phthalic anhydride, are used to increase the glass transition temperature (*T*_g_), chemical resistance, and hardness of PUs. Furthermore, IPA is used as a principal aromatic diacid in coating applications [112]. However, these acids are petroleum-based chemicals, and thus, cause environmental degradation. Meanwhile, adipic acid, 1,4-cyclohexanedicarboxylic acid (1,4-CHDA), and succinic acid are used as aliphatic diacids in biodegradable polyol production. Adipic acid is the principal aliphatic acid for preparing polyester polyol via ringopening polymerization. However, 1,4-CHDA exhibits excellent aliphatic and aromatic diacid characteristics. 1,4-CHDA also demonstrate rapid reaction; maintained hardness and flexibility balance; resistance to chemicals and humidity low resin color, improved solubility in methyl ethyl ketone, and stable hydrolytic polyol properties.

Meanwhile, bio-succinic acid is a 100% bio-based diacid used to synthesize polyester polyols. This acid has 90% and 50% carbon footprint with respect to petro-based adipic acid and phthalic anhydride, respectively. It can provide higher glass transition temperature and slightly higher viscosity with saturated polyester resins. Succinic acid-based materials are renewable, biodegradable, and are also used to synthesize UV-curable acrylates with commercially available bio-based acrylic acid [105].

### 2.3. Nano-Scale Materials

Nano-scale materials such as nanotubes, nanorods, nanowires and nanoparticles have been increasingly applied in nanotechnology and contribute as value-added materials in bio-based composite fabrication. Nano-scale materials have altered the properties and applications of nanocomposites because of their unique physical and chemical characteristics. The significance and type of the nano-scale material depend on the purpose of the nanocomposites. There are two major types such as inorganic and organic materials that have been used as nanoparticles in the field of bionanocomposite fabrication in recent years. On the one hand there are nano-scaled titanium oxides or cerium oxide doped with silica, alumina, gold, ZnO, and ZnO/SiO_2_, TiO_2_, GeO_2_, Cu_2_O, Cr_2_O_3_, Fe_2_O_3_, PbO_2_, CaCO_3_, CdS, Ag, Pt, and Pd as the inorganic materials used in many applications, especially in the biomedical and tissue-engineering fields [113]. Not only the amount of nanoparticles but also their shape influence the properties of the bionanocomposite materials and are thus reviewed briefly in this paper. Furthermore, there are several nanoparticles such as polystyrene nanosphere, functionalized polystyrene nanosphere, fluorescent nanosphere, nanoparamagnetic particles, and coated polystyrene nanoparticles that are commercially available. Organic materials such as natural fibers, organic liquids, nano-scale cellulose crystals, graphene, and carbon nanotubes are common materials used as fillers in bionanocomposite coating film fabrication. Several studies have mentioned a few versatile methods to combine chitosan with various negatively charged hydroxyapatite microparticles, clay nanoparticles [114] and, most recently, graphene oxide and carbon nanotubes. These versatile methods can be adopted to fabricate free-standing coating films and hydrogels. Saponite is a natural mineral that is also used as an nano-scale material in the form of nanoplatelets to fabricate chitosan-based nanocomposite films [114].

## 3. Bionanocomposite Coating Films

A nanocomposite is characterized by one or more discontinuous phases distributed in one continuous phase. The continuous phase is called the matrix, whereas the discontinuous phase is called the reinforcement or reinforcing material. In the discontinuous phases, one of the phases with components has at least one dimension that is approximately 10^−9^ m. Nano-scaled materials, biopolymers, and cross-linkers act as legitimate members in bionanocomposite fabrications. Moreover, the synthetic routes of bionanocomposites are exploited in three fields: chemical functionalities, self-assembly of copolymer monomer control synthesis, and nanoparticles. Several conventional and novel methods have been reported recently for preparing bionanocomposite coating films, such as suspension-casting, water evaporation, hot pressing, and pressure extrusion [113]. Excessive water evaporation is difficult to manipulate using the suspension-casting and water-evaporation methods. These methods are also typically time consuming, and may take several hours or even a few days [115]. Alternatively, a recent study suggested that the pressure extrusion method could be used to overcome this issue under ambient temperature.

**Figure 7:**
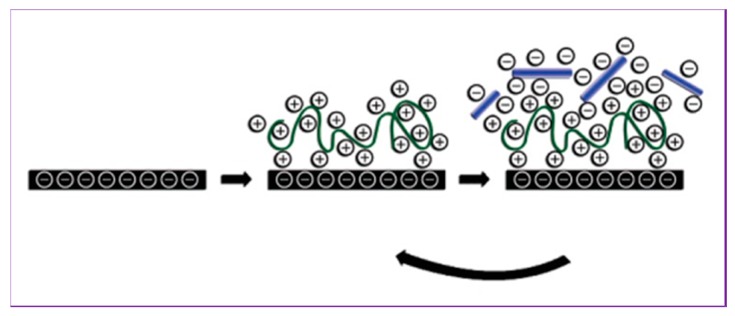
Schematic illustration of the build-up of electrostatically adsorbed multilayered films. Reprinted (adapted) with permission from [117], copyright (2016) American Chemical Society.

Monolayer and multilayer films are the two major categories of coating films. A monolayer film can be used in coating technology to improve unique properties by modifying the surface of the other materials. On the other hand, multilayer films are composed layer-by-layer formation technology. In this technology, hydrogen bonds and electronic interactions act as driving forces between two layers, which are deposited via solution-dipping or spin-coating. However, as driving forces, non-electrostatic interactions exert an influence when layer-by-layer is assembled for two bio-based materials. De Mesquita et al. [116] also used the layer-by-layer technique to prepare chitosan and CNC-based nanocomposite films as alternative bio-based materials. Poly-(diallyldimethylammonium chloride) and poly-(allylamine hydrochloride) (PAH) have also been used as layer materials incorporated with CNC in the layer-by-layer assembling technique [117], as shown in Figure 7.

### Cellulose-Based Nanocomposite Coating Films

Cellulose is used in many applications because of its outstanding properties, including stiffness, strong reinforcement in composite, excellent dimensional stability [118], water-absorption capability in the food industry, [119] and mechanical properties. Cellulose coatings have received widespread attention in the laboratory because of their use in high-performance nanocomposite films with low environmental impact [120]. These coating films may be used in optical [121], biomedical [122,123], medical sensor [124,125], or electronic applications [126]. The elastic property of cellulose-based materials has been reported. These materials exhibit different values with respect to their composition nature in plant materials. The elastic modules of bulk natural fiber such as wood present 10 GPa. Chemical modification has been considered an essential method to endow materials with several unique features [125,127].

Given the large surface area and enhanced hydrogen-bonding capability of fibrils, pre-chemical treatment is typically conducted to destroy the hydrogen bond and to easily conduct chemical modification [128]. Chemically modified cellulose is widely used in various applications, particularly in coating film applications. Cellulose acetate is the first chemically modified cellulose used for filter membranes in water purification [129]. Cellulose acetate has several reliable properties such as high biocompatibility, good desalting properties, moderated hydrophilicity, and high potential flux [130]. However, cellulose acetate has poor fouling resistance caused by the accumulation of biological foulants, such as bacteria and protein cells, on the membrane surface [131]. Nitrocellulose is another form of chemically modified cellulose and is used in the paint industry as an alternative derivative for traditional lacquer and coatings because of the abundance of their raw materials, industrial maturity, and biodegradation. These alternative derivatives overcome increasing indoor and interior environmental [132] and volatile organic compound emission issues [133].

Other chemically modified cellulose derivatives, such as hydroxypropylmethyl cellulose, carboxymethyl cellulose, and methyl cellulose, are obtained by deriving the hydroxymethyl groups at positions 2, 3, or 6 of the hydro-glucose residues. These derivatives exhibit improved solubility and are used in fibers, films, and gel-based materials [134]. Carboxymethyl cellulose can irreversibly bind with positively charged chitosan via complex formation [26]. Cellulose derivative-based films function as effective barriers against O_2_/CO_2_ and demonstrate good tensile resistance [135,136]. However, given the hydrophilic nature of cellulose, the prepared cellulose-based edible films do not function as an efficient water-vapor barrier [136,137]. Furthermore, adding the prepared CNCs from microcrystalline cellulose via sulfuric acid hydrolysis to PLA or PLA-polyhydroxybutyrate films improve their thermal stability and water permeability [138].

Cellulose sulfate is another type of chemically modified cellulose that is prepared by partial or complete substitution of the 6-hydroxyl groups with sulfate groups. The cellulose sulfate-based films exhibit poor water-vapor barrier capability because of their excellent solubility behavior [139]. However, given the 40% usage of the petroleum-based chemicals in the packaging industry, this alternative cellulose sulfate is considered a renewable alternative source to conventional petroleum-based materials [140]. Cellulose sulfate-based nanocomposite films that incorporate with other molecules such as lipids, glycerols, and oleic acid have extended their application in the field of packaging films. Cellulose sulfate-based hydrocolloid films combined with lipids exhibit better functionality than pure cellulose sulfate films. Glycerol is one of the most popular plasticizers used in cellulose sulfate films to ensure its stability and compatibility with hydrophilic bio-polymers [141].

In cellulose sulfate-oleic acid composite film, oleic acid decreases water content in the emulsion as the intermolecular distance of cellulose sulfate increases. Alkyd resin modified by coconut oil or soybean oil can significantly improve the mechanical properties of coating films; in particular, it reduces the brittleness of carboxymethyl cellulose nitrate films [142]. This type of resin is typically used as an auxiliary film-forming material. Alkyd resins also exhibit good consistency with water.

## 4. Application of Bionancomposite Coating Films

In general, the applications of bionanocomposite coating are ubiquitous in various applications such as in tissue engineering, biomedical, glass coating, food packaging, wood coating, steel coating, drug coating, and fruit coating. However, each coating application has several purposes depending on the demands of the final outcome.

### 4.1. Biomedical Application

Collagen is a biopolymer widely used in medical application, particularly in the fermentation of tissue and organs. Collagen coatings on Ti hard-tissue implants are widely reported in recent works, for their stimulating cellular response [143], increasing remolding [144] that improves bone growth, and bone-implant contact [145]. Collagen is used as film, disk, sheet, shield, sponges, gel, hydrogel and pellet in the drug-delivery system. In biomedical applications, reviews only consider the films’ characteristic of the collagen biopolymers. Collagen films have been used for the treatment of tissue infections, specifically infected corneal tissues or liver tissues. Collagen is mainly applied as a membrane with 0.01–0.5 mm film thickness [146]. As the composite matrix, collagen can be combined with a recombinant human morphogenetic protein-2 (rhBMP-2) for implant-bone formation [147]. Nakagawa et al. [148] reported that a collagen-based drug-delivery system (BMP-2 loaded collagen matrix) is highly efficient as a biological onlay implant.

Alternatively, collagen films and disks as gene-delivery systems have many advantages, including isolation of transplanted cells from the host immune system [149] and long-term delivery of therapeutic transgenic product. Collagen sheets are evaluated as a local delivery carrier for cancer treatment; the etoposide (VP-16)-loaded collagen sheets as anti-cancer agents have been revealed to maintain the drug at the target site for a long period [150]. The biodegradable collagen films are not only severed as scaffolds for the survival of transfected fibroblasts, but the composite of collagen and elastin are also suitable for many potential medical applications in reconstructive and plastic surgery [151].

Composite collagen films incorporated with elastin have been reported with various monomer ratios; accordingly, almost consistent tensile values were observed [152]. Tensile strength can be used to evaluate the mechanical strength, resilience activity, endurance, and biocompatibility of the biomaterials. Collagen sponges have been fabricated via isolated pure collagen from bovine skin, followed by swelling at pH 3.0, which are extremely useful for many types of wounds, mostly severe burns. Furthermore, collagen sponges are a highly efficient material for the recovery of skin and various types of artificial skin incorporation of gelatin [153].

### 4.2. Tissue-Engineering Application

A number of natural and synthetic biodegradable polymers are studied in tissue engineering. Pullulan, collagen, chitosan, PHA, PGA, and PLA are used in several tissue-engineering applications, including tissue replacement, bone substitutes, membrane, engineered tissue, scaffolds, guided tissue regeneration, reinforcement and support for weak tissues [154,155]. Carboxymethyl pullulan and its conjugation with heparin can inhibit the proliferation of smooth muscle cells in vitro [156]. A cellular bilayer artificial skin was fabricated by Suzuki et al. [157], in which they used silicon as the outer layer and inner collagen sponges. Results showed good long-term postoperative appearance of the split thickness skin graft site. Liu et al. [158] studied chitosan-based bionanocomposite films incorporated with halloysite nanotubes as scaffold materials in tissue engineering. The resultant nanocomposite films exhibit a cytocompatible nature with maximum loading of 10% halloysite nanotubes. Furthermore, Kim et al. [159] studied the multi-tissue-engineering applications of chitosan-based bionanocomposite. Hajiali et al. [160] used salt-leaching technology to prepare PHA-based nanocomposite scaffold for bone tissue engineering, in which 10% bioglass nanoparticles were incorporated. Results showed 84% porosity of the scaffold structure. Moreover, the contribution of synthetic biopolymers and their nanocomposites in tissue-engineering applications have been reviewed by Okamoto et al. [161].

### 4.3. UV Protection

UV radiation is harmful to all living things. Researchers and industries have fabricated various UV ray-protecting products such as sunblock cream and lotions, sunglasses, hats, window protectors and arm protectors and clothes, including rash guards and swimming T-shirts. UVB, which is defined within the range of 315–280 nm, is absorbed by the ozone layer; however, a range of rays generated by UVB reaches the surface of the Earth. UVA has a longer wavelength of 315–400 nm, and its rays reach the surface of the Earth without any deterrent [162]. UV rays can damage the skin through short- and long-term effects. Sunburn, tanning, and photosensitivity (a disease called porphyria caused by UV rays) are considered short-terms effects, whereas long-term effects are highlighted by skin and eye cancer, freckles, solar brown spots (lentiginous), and melanocytic nevi (moles). However, materials used in sun-protection products for specific purposes have commercially high costs and contain non-biodegradable substances.

The sun protection factor (SPF) is one of the important parameters used to measure the UV-blocking capability of materials. This factor, which was introduced by Franz Greiter in 1962, has a unit of mg/cm^2^ [163]. On the basis of UV-blocking research on plant materials, high SPF values have been given to *Menta piperita* leaf and *Lycopersicom esculantum* fruit that are 8.184 and 5.998, respectively. These plant extracts have significant capability to absorb the UVA (400–315 nm) and UVB (315–280 nm) regions of the UV rays [164]. UV absorbers play a major role in the UV-protecting property of materials. In such materials, the absorbers act as scavengers or singlet oxygen quenchers, and convert the electronic excitation energy into thermal energy. That is, absorbers are excited when they are hit by high-energy short-wave UV radiation and move to a higher energy state. Consequently, the absorbed energy may be dissipated as longer-wave radiation [165]. Several inorganic and organic colorless compounds have been used as UV absorbers in textiles and sunblock lotion. These compounds include 2-ethylhexyl-4-methoxy-cinnamate with high refractive index [166], *o*-hydroxyl benzophenones, *o*-hydroxyphenyl triazine, and *o*-hydroxy phenyl hydrazine. Furthermore, benzotriazole, hydro benzophenone, and phenyl triazine are primarily used as UV absorbers for coating applications [165].

Inorganic composite materials and compounds also exhibit UV-blocking capability and have been studied in recent years. UV absorbers have been developed for coatings based on the composition of inorganic particles such as titanium oxides or cerium oxide-doped with silica, alumina, organic liquids, iron, ZnO, and ZnO–SiO_2_. Their nano-scale shapes also influenced the UV-blocking process. Dumbbell-shaped ZnO nanorods improve coating application in the textile industry and exhibit wider UV-blocking range (400–280 nm), whereas the UV-blocking range of ZnO nanosols and ZnO nanorods are within the range of 352–280 nm and 375–280 nm, respectively. However, a decreasing trend of UV transmittance is observed in cotton textiles coated with ZnO–SiO_2_ nanorods [167].

Ching et al. [168] developed the PU/nanosilica composite as a surface protective coating layer for polyethylene greenhouse films. They observed excellent thermal stability with decreasing photo degradation of the composite coating compared with polyethylene that uses 6 wt % nanosilica. Cotton-based cellulosic fibers and lignins have certain levels of UV-absorbing capability and are used in natural organic coatings [31]. Several studies have reported UV-absorption capability in textile with respect to their nano-scale shape. For example, needle-shaped ZnO nanorods have the capability to block the UV-transmission of a part of the UVA region (370–315 nm) and the entire UVB region (315–250 nm) [169]. Flower-like ZnO fabricated on cotton cellulose has high absorption capability at a wavelength of 350 nm [169]. Anatase is one of the three mineral forms of titanium dioxide; it provides UV-blocking capability within the range of 332–280 nm. Textile industries use these materials to enhance the quality of their products with UV-blocking capability.

In the last few decades, researchers have focused on extracting nanocellulose from natural sources, such as cotton, wood, algae, bamboo, sisal, and bacteria because of its high strength, large surface area, and unique optical properties [170]. Nanocellulose-based films exhibit excellent optical transparency, which decreases light scattering within a large portion of light transmittance. Furthermore, the properties of functionalized nanocellulose-based films exhibit improved suitability in their applications such as UV-sensitive polymers, clean windows, contact lens, car windshields, and special biological test containers. These films also have an extraordinarily low thermal expansion coefficient with a range of 12–28.5 ppm·K^−1^. Nanocellulose-based films are easier to process at high temperatures than plastic substances and possess high transparency, as well as outstanding UV-blocking and biodegradable properties [113]. UV-blocking capability has also been studied on several plants with respect to their parts, including the leaves of *Menta piperita*, *Azadiracha indica*, *Oscimum sanctum* and *Aloe vera*; the fruits of *Lycopersicom esculantum* and *Carica papaya*; and the flowers of *Rosa damascene*, *Crossandra infundibuliformis*, *Tagetus patula*, and *Tagetus erecta.* A recent study has found that untreated bamboo viscos fiber possesses inherent UV protective properties and demonstrates minimal antimicrobial activity [171].

The nanocomposite nanocellulose films with ZnO nanomaterials exhibit better UV-protectiong, transparency, and sensitivity capabilities. However, given the high water-binding capacity of nanocellulose, dewatering difficulties and nanocellulose hybrid heterogeneous architectural issues remain during the production process of nanocellulose-based films [113]. Meanwhile, the excessive use of UV-absorbing nanoparticles in textiles is causing ecological problems as a result of the existence of these materials in effluent solutions.

Lignin-based bionanocomposites have significant adhesive [172] and biodegradable properties, and are used as stabilizing agents in aqueous alumina and ceramic suspensions [173]. Lignin acts as a source of aromatic chemicals [174] in PU. Lignin-based nanocomposite coating films are prepared using CNCs and are used in various applications such as medical, biological, optical and sensors, and electronic.

Lignin inherently possesses UV-absorbing capacity [31] and thus, lignin has been used in coating applications with suitable cellulose-to-lignin ratio. The results of UV absorption, transparency, and colorlessness coating have been obtained without the chemical modification of cellulose and lignin fractions. However, novel chemically modified lignins (industrial lignin) [175] such as lignosulfates, kraft lignin, and acetylated lignin have been recently developed [176]. These types of lignins are used for coating applications incorporated with cellulose fiber, commercial derivatives, or nanocellulosic polysaccharides to improve the mechanical resistance, hydrophobicity, and oxygen barrier properties of the fabrication materials.

**Figure 8:**
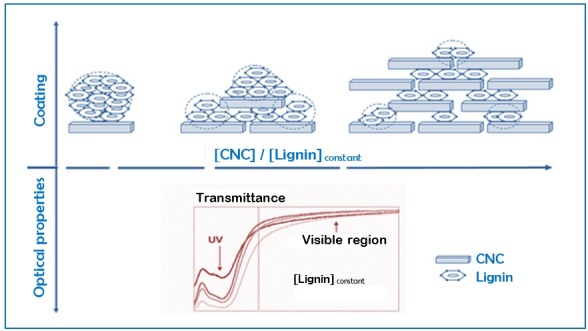
Illustration of the structural arrangement of CNC/lignin nanocomposite coatings and UV-transmittance. Reprinted (adapted) with permission from [31], copyright (2016) American Chemical Society.

CNC-lignin composite coating films are more homogeneous than pure synthetic lignin coating or microcrystalline-lignin coating. These films exhibit various spectroscopic properties in the UV/Visible spectrum. Given the covalently bonded phenolic acid moieties in lignin, CNC-lignin coating films exhibit anti-UV properties that extend up to 340 nm, as illustrated in Figure 8. The properties of these coating films are reported as natural, organic UV absorbent, and visible transparent coating. Furthermore, the mechanical resistance of CNC and the antibacterial properties of lignin are opened for investigation [31].

### 4.4. Antifouling Application 

Cellulose acetate-based membrane surface incorporating with polysaccharides has been fabricated using the layer-by-layer (LBL) technique to study antifouling application. The positive and negative charge functionalities of polysaccharides [177] have been utilized in this type of coating given their diverse chemical compositions [178].

Chitosan and carboxymethyl cellulose on cellulose acetate membrane have also been reported and applied to reduce bovine serum albumin fouling on the membrane [179,180]. The layers can be detached by high ionic strength and low pH. This phenomenon demonstrates the reliability of these substances as removable hydrophilic functional coatings for cellulose acetate. However, the protein rejection behavior of this membrane has not been studied extensively.

### 4.5. Food Preservation Applications

Synthetic polymers are used in ubiquitous food packaging applications where they provide permeability to microbial, chemical, organic vapor and oxygen from the environment and allow product display while biopolymers are notorious for their higher water-vapor permeability [181]. An exfoliation of nanoclay (montmorillonite) into polymers creates a maze structure that presents a tortuous path to moving gases, greatly slowing their permeation rate.

Hydroxyl propyl methyl cellulose (HPMC) has been found to be a promising material for edible coatings or films for packaging by Burdock et al. 2007 [182]. Incorporation of chitosan nanofiller into the HPMC matrix improves the mechanical properties and water-vapor permeability while significantly reducing oxygen permeability. Consequently, HPMC-chitosan nanofiller composite films provide potential properties and thus become promising materials for food packaging with better shelf-life [183]. Soybean protein nanocomposite films provide an improved elastic modulus and tensile strength with reduced water permeability under ultrasonic treatment with the incorporation of MMT [184].

The compatibility of rosemary oil with chitosan/montmorillonite (MMT) nanocomposite has been confirmed to produce an active bionanocomposite for food packaging. The good interaction between chitosan and MMT can improve the water-vapor permeability, water sensitiveness, and mechanical properties of chitosan films. In addition, chitosan nanocomposites containing rosemary essential oil exhibit antimicrobial properties and high phenol content. These properties make chitosan highly interesting for food preservation.

### 4.6. Water-Vapor Barrier

Packaging plays an important role in the production, transportation, and storage of food or pharmaceutical products. Such products require a material with a high moisture-barrier capacity and potential biodegradability. The common approach to improve the moisture-barrier capacity of nanofibrillated cellulose films for packaging applications is to treat the surfaces of nanofibrillated cellulose substrates with synthetic or bio-based polymers (e.g., whey proteins, PLA, PCL, beeswax) [185,186], or inorganic impermeable particles such as mica [187] or MMT [188] through laminating extrusion, vacuum deposition, and multilayer coating technologies [189,190]. Acetylated epoxidized soybean oil is an interesting polymeric material obtained from renewable natural resources. This material contains acrylate functional groups to polymerize/copolymerize easily via free-radical reaction under several initiator systems. Pure acetylated epoxidized soybean oil polymer behaves similarly to an amorphous cross-linked rubber, which fails to produce suitable shapes and to provide high mechanical properties [191] by itself. However, this polymer is expected to have a good moisture barrier-capacity because of its good film-forming and hydrophobic properties.

Several studies have indicated that through controlled polymerization or co-polymerization with other chemical species, this material is able to provide better polymers with optimized properties. These polymers can be used extensively as surface coatings and adhesive agents [192]. To date, acetylated epoxidized soybean oil in cellulose films or fiber networks in packaging production has seldom been used. The film-forming mechanism is attributed to acetylated epoxidized soybean oil, cellulose, and 3-aminopropyltriethoxysilane as constituent nanocomposite materials. Water-vapor transmission rate has been studied with varying of 3-aminopropyltriethoxysilane contents. With a low content (10 wt % of acetylated epoxidized soybean oil), the reduction in water-vapor transmission rate is insignificant. A continued increase in 3-aminopropyltriethoxysilane content leads to a reduction in water-vapor transmission rate value and to an increase in film hydrophobicity (high contact angle). The best result appears at 3-aminopropyltriethoxysilane 30 wt %, and the resulting water-vapor transmission rate is as low as 1714 g/m^2^ at 24 h. Furthermore, the increase in 3-aminopropyltriethoxysilane content does not result in any remarkable reduction.

## 5. General Properties of Bionanocomposite Coating Films

Several studies have been reported on the improvement of the properties, including mechanical properties, barrier properties, functional properties, water solubility [193,194], and thermal stability [1,22] of natural polymer-based bionanocomposite films incorporated with nanoparticles. Furthermore, the combination of antimicrobial/antioxidant compounds, such as essential oils or any natural agents and nanoclay in chitosan film results in acceptable structural reliability and barrier properties. This finding is attributed to the nanocomposite having no combined effect of nanoclay and antimicrobial/antioxidant compounds. For example, chitosan-based nanocomposite with MMT clay particle and rosemary essential oils as antimicrobial/antioxidant compounds exhibit good mechanical properties, water sensitiveness, and improved water vaporization properties [195].

The inherent properties of fabricated bionanocomposite coating films are significant for its applications. Mechanical, non-mechanical, and thermal properties are regarded as the major properties of bionanocomposite coating films. However, the physical and chemical characteristics of the constituent materials of bionanocomposite coating films can significantly alter these properties. Mechanical properties do not only depend on the morphology and dimension of the constituent materials, such as nanoparticles and polymer matrix, but also on the formation of the percolating whisker network [196]. The percolation of the whiskers depends on the aspect ratio of the filler, such as sisal nanowhiskers with high aspect ratio. In particular, a filler in polyvinyl acetate-based nanocomposite films can improve mechanical and thermal properties at low fiber loading [196].

Tunicin whiskers with a high aspect ratio as filler constituents of amorphous poly(β-hydroxyoctanote) (PHO) lead to high mechanical properties of the composite [197]. Similarly, carbon nanotubes with a high aspect ratio as filler exhibit high mechanical properties of the nanocomposite films [198]. Mechanical properties such as tensile strength, elongation at break, and Young’s modulus of novel nanocomposite films and the applications of these films are presented in Table 4. Young’s modulus linearly increased with microfibrillated cellulose loading by up to 40 wt % using a phenolic resin [199]. The different types of polymer matrix with the same microfibrillated cellulose loading have shown different values of tensile strength and Young’s modulus. For example, the tensile strengths for microfibrillated cellulose with phenol formaldehyde and starch matrix are 370 and 160 MPa, whereas their Young’s modulus values are 19 and 6.2 GPa, respectively [200].

Non-mechanical properties are also key players in bionanocomposite coating films. These properties mainly include transparency, optical property, flexibility, a light weight, resistance to moisture, adhesion to the substrate, thickness, water-vapor permeability, surface hydrophobicity, oil-holding capacity, chiral nematic and conductivity, antimicrobial activity, UV-blocking properties, and barrier properties. Transparency and optical properties are value-added properties of the bionanocomposite coating films when they are considered to be applied as coating films for auto mobiles and aerospace vehicles as well as for UV-protective window applications. Microfibrillated cellulose has been used with resins in a recent study to fabricate coating films with high transparency; this process can be conducted by eliminating surface scattering. Wood-cellulose-based nanocomposite has also been used as an optically transparent substrate for flexible organic light-emitting displays [201]. Furthermore, an optical transparency analysis has been performed by Ching et al. [202] for a PVA/nanocellulose composite incorporated with nanosilica as a reinforced material. They found a decreasing trend of visible light transmission with an increasing wt % of nanosilica.

## 6. Conclusions

Bio-based nanocomposite coating films have been used in numerous recent applications with different aspects because of environmental factors and their biodegradability properties. Several recent studies focused on improvement strategies to enhance the quality of bionanocomposite coating film materials. Several naturally occurring low-cost bio-based materials have been on the track to fabricate these coating films through conventional and novel film-producing technologies. These approaches have improved the field of applications as well as the physico–chemical properties and mechanical properties of bionanocomposite coating films. In particular, these approaches have also been active in natural bio-polymer reinforcement, scaffold fabrication, implant device manufacturing, drug delivery and packaging technology.

The future of these coating films does not only depend on the constituent materials, including the scalability of nanoparticles and the stoichiometric ratio of biocomposites, but also on the field in which these films are applied. Furthermore, the future outlook for developing novel coating films with enhanced quality is promising.

## Figures and Tables

**Table 1:** The mechanical properties of plant fibers with an increasing order of tensile strength [8].

Fibers	Density	Elongations at break (%)	Young’s modulus (GPa)	Tensile strength (GPa)
Coconut	1.15	15–40	4–6	131–175
Bamboo	0.6–1.1	–	11–17	140–230
Kenaf	1.2	1.6	14–53	240–930
Cotton	1.5–1.6	7–8	5.5–12.6	287–597
Flax	1.54	1–4	27.5–85	345–2000
Sisal	1.45–1.5	2–7	9–22	350–700
Hemp	1.47	1.6	17–70	386–800
Jute	1.44	1.5–1.8	10–30	393–773
Ramie	1.5–1.56	1.2–3.8	27–128	400–1000
Nettle	1.51	2.1–2.5	24.5–87	560–1600
E-glass	2.5	2.5	70	2000–3500
Carbone	1.4	1.4–1.8	230–240	4000

**Table 2:** Chemical modification of polyhydroxyalkanoate surface via different synthetic routes.

Functional groups	Synthetic rout	Reaction condzition	Chemically modified PHA	Ref.
Mono-hydroxyl	Transesterification via acid catalyst	H_2_SO_4_, MeOH CH_2_Cl_2_, 100 °C	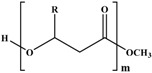	[66]
Di-hydroxyl	Transesterification via acid catalyst	1,4-Butanediol APTS (para-toluene sulfonic acid monohydrate) CHCl_3_, 60 °C	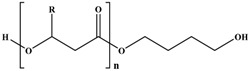	[67]
	Transesterification via Dibutylene dilaurate catalyst	Ethylene glycol Diglym, 140 °C Dibutylene dilaurate	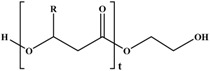	[68]
Branched-poly(ethyleneimine)	Grafting via Michael addition	CHCl_3_ 45–50 °C	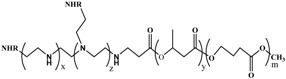	[69]
Carboxylic acid	Oxidation	Osmium tetroxide Oxone, BuOH, DMF 60 °C, 8 h	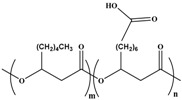	[70]
Epoxidation	Oxyrane addition	*m*-Chloroperbenzoic acid, CHCl_3_ 20 °C, 12 h	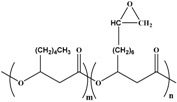	[71]

**Table 3:** Protein films’ incorporation of nanomaterials with their properties and applications.

Protein type	Nano-material	Film properties	Application	Ref.
**WPI**	TiO_2_ < 1 wt %	Transparent Oxygen barrier Improved antimicrobial properties	Industrial: Edible film Coating and packaging material	[82]
	<0.25 wt %	Promoted florescent and TS	Industrial: Food and cosmetics	[83]
	>0.25 wt %	Fluorescent quenching Reduced TS, WVP, Elongation and light transmittance		
**Soybean Protein**		Poor response for moisture High rigidity	Industrial: Packaging material	[84]
	MMT 5%–15%	Reduce WVP Improved MP, T_g_, DMP At pH = 9 and 5 wt % of MMT	Industrial: Packaging material	[85]
**Zein**	Kaolin	WVP decreased by 50 wt % Kaolin Reduce oil permeability	Industrial: Barrier coating material for paper and paper board	[86]
**Collagen**	HA 10 wt %–30 wt %	Lowest contact angle 36.5 High proliferation rate at 20 wt % of HA	Medicinal: Implant	[87]
**Fish gelatin**	MMT 0 wt %–9 wt %	Improved WVP TS and Elongation maximum at 5 wt % of MMT	Industrial: Food	[88]
**Egg albumen**	–	Improved switching properties	Industrial: Nonvolatile memory application (memristor device)	[89]
**Keratin**	Graphene Oxide 0.1 wt %–0.5 wt %	Increased storage modulus up to 200 °C at 0.1 wt % of graphene oxide	Material fabrication	[90]

WVP: Water-vapor permeability; MP: Mechanical properties; DMP: Dynamic mechanical properties; MMT: Montmorillonite; TS: Tensile strength; HA: hydroxyapatite; WPI: Whey protein; *T*_g_: Glass transition temperature.

**Table 4:** Comparison of mechanical properties of various bionanocomposite films at maximum load. (*) indicating the unavailability of the relevant data.

Constituent materials of composites	Properties				Applications	Reference
	CAL/(°) ^a^ WVP/(10^−10^ g/ms·Pa) ^b^	Elongation (%)	Young’s modulus/(MPa)	Tensile strength/(MPa)		
SPI/EDGE/CNC	53–54 ^a^	86–87	48–50	4–6	Preliminary	[11]
SPI/EDGE/MCNC	56–57 ^a^	78–80	65–70	5–7	Preliminary	[11]
CH/MAC-CNC	*	8–11	3500–3700	105–108	Preliminary	[203]
CH/CNC-Gly	44–45 ^a^	5–6	351–500	8–11	Special	[204]
CH/Gly-OO	59–60 ^a^	11–12	239–420	10–13	Special	[204]
PVA/GR-CNC	*	79–85	1432–1550	36–38	Reinforcement	[205]
CH/C-AgNPs	*	*	*	*	Antimicrobial	[206]
CNC/ZnO	88–90 ^a^	40–45	6350–7000	55–56	Preliminary	[207]
CA/CNC	*	16–18	95–1000	30–33	Preliminary	[208]
PVA/CNWs/CH	*	126–150	*	62–65	Food packaging	[209]
AG-CNC/SEO	2–3 ^b^	51–55	46–50	20–22	Packaging	[210]
CMC/ST-CNC	4.8–5.2 ^b^	19–20	1650–1670	110–116	Packaging	[211]
CH/MMT/REO	0.35–0.50 ^b^	4–5	*	74–79	Preliminary	[37]
SM/KA	*	28–32	136–139	5–6	Food Packaging	[212]
CS/Gly/OA	94–96 ^a^, 15–16 ^b^	6–7	*	34–36	Packaging	[213]
Chi/CH-TA	*	20–25	1300–1400	50–55	Preliminary	[214]
CH/CNC-TA	*	15–25	1700–1750	55–60	Preliminary	[215]

CAL: Contact angle at (0) time indicating as “a”; WVP: Water vapor permeability indicating as “b”; SPI: Isolated soybean oil; EDGE: Ethylene glycol diglycidyl ether; CNC: Cellulose nanocrystal; MCNC: Modified CNC; CH: Chitosan; MAC: Methyl adipoly chloride; Gly: Glycerol; OO: Olive oil; PVA: Polyvinyl alcohol; GR: Stabilized graphene; C: Cellulose; Ag: Silver; NPs: Nanoparticles; ZnO: Zinc oxide; CNWs: Cellulose nanowhiskers; AG: Agar; SEO: Savory essential oil; CMC: Carboxymethyl cellulose; ST: Starch; MMT: Montmorillonite; REO: Rosemary essential oil; SM: Semolina; KA: Kaolin; CS: Cellulose sulfate; OA: Oleic acid; Chi: Chitin; TA: Tannic acid.
